# Multi-Defect Detection in Additively Manufactured Lattice Structures Using 3D Electrical Resistance Tomography

**DOI:** 10.3390/s22239167

**Published:** 2022-11-25

**Authors:** Yening Shu, Saptarshi Mukherjee, Tammy Chang, Abigail Gilmore, Joseph W. Tringe, David M. Stobbe, Kenneth J. Loh

**Affiliations:** 1 Department of Structural Engineering, University of California San Diego, La Jolla, CA 92093, USA; 2 Active, Responsive, Multifunctional, and Ordered-materials Research (ARMOR) Laboratory, University of California San Diego, La Jolla, CA 92093, USA; 3 Lawrence Livermore National Laboratory, Livermore, CA 94550, USA

**Keywords:** 3D printing, absolute imaging, damage identification, ERT, nondestructive evaluation, sensitivity matrix

## Abstract

Cellular lattice structures possess high strength-to-weight ratios suitable for advanced lightweight engineering applications. However, their quality and mechanical performance can degrade because of defects introduced during manufacturing or in-service. Their complexity and small length scale features make defects difficult to detect using conventional nondestructive evaluation methods. Here we propose a current injection-based method, electrical resistance tomography (ERT), that can be used to detect damaged struts in conductive cellular lattice structures with their intrinsic electromechanical properties. The reconstructed conductivity distributions from ERT can reveal the severity and location of damaged struts without having to probe each strut. However, the low central sensitivity of ERT may result in image artifacts and inaccurate localization of damaged struts. To address this issue, this study introduces an absolute, high throughput, conductivity reconstruction algorithm for 3D ERT. The algorithm incorporates a strut-based normalized sensitivity map to compensate for lower interior sensitivity and suppresses reconstruction artifacts. Numerical simulations and experiments on fabricated representative cellular lattice structures were performed to verify the ability of ERT to quantitatively identify single and multiple damaged struts. The improved performance of this method compared with classical ERT was observed, based on greatly decreased imaging and reconstructed value errors.

## 1. Introduction

Lattice structures are bio-inspired 3D configurations of repeated and open unit cells defined by interconnected struts and nodes [1,2]. Relative to conventional bulk materials, topologically ordered lattice structures can exhibit impressive mechanical strength, stiffness, thermal, and electrical properties while using significantly less material. In other words, they possess higher strength- and stiffness-to-weight ratios. These advantages have led to their broad applications in advanced lightweight naval, automobile, aerospace, and other engineered structures [3,4,5,6].

Increasing performance demands for these ultra-lightweight engineering applications means that cellular lattice structures need to be fabricated with greater complexity and with smaller feature sizes. Conventional manufacturing processes, such as wire weaving [7], high-temperature forming and diffusion bonding [8], and the interlocking method [9], are unsuitable and too time-consuming for fabricating lattice structures with complex nodal connections. Recent advances in additive manufacturing (AM) have enabled methods to realize cellular lattice structures with intricate geometries [10,11]. Some of the widely used AM methods include fused deposition modeling [12] and stereolithography [13] for polymer-based structures, as well as extrusion [10], powder bed fusion [14], and direct ink write [15] for metallic cellular lattice systems. 

Despite the ability to use AM to fabricate complex cellular structures, their functional performance strongly depends on manufacturing quality. The presence of minor defects could compromise the structural integrity of the entire part [16]. For instance, nozzle clogs, micro-voids, and pores that occur during extrusion or uncontrolled thermo-mechanical behavior in powder bed fusion may induce cracks, shrinkage, uneven surfaces, and nodal disconnections in the struts [17]. During storage, transit, or use, these weakened struts are prone to stress concentrations, which can lead to defect propagation, broken struts, and partial or complete lattice structure failure [18]. Therefore, quality assurance and control of AM parts require that the type of defects and damage locations be identified whether they are incurred during manufacturing or when in service. 

Traditional nondestructive evaluation (NDE) methods, such as x-ray computed tomography (CT) and ultrasonic measurements, have been used to detect defects such as voids and inclusions; however, they can be inefficient for inspecting complex cellular lattice structures. For example, CT reconstruction of defects in lattice structure struts requires multiple projection slices and can be computationally intensive, slow, and expensive [19]. Similarly, ultrasonic testing requires a dense array of transducers and complicated wave generation and propagation patterns to evaluate the different scales and locations of defects [20]. Another approach is to physically integrate sensors as part of lattice structures for continuous monitoring even during post-production. An example is the printing of conductive paths for capacitive sensing or radio-frequency identification (RFID) [21]. However, the sensing area for these approaches is restricted by the limited number of paths and may not be suitable for NDE of the entire lattice structure. 

This study aims to overcome these limitations by leveraging 3D electrical resistance tomography (ERT) to directly detect and accurately identify the locations of damaged struts in cellular lattice structures. ERT aims to reconstruct the conductivity distribution of a conductive target that is directly correlated to damage or strain states by using only boundary electric potential measurements [22,23]. The utilization of a target’s electromechanical properties exempts inspection from complex operations (i.e., multiple projections of CT) [22,24]. 

A major challenge when using electromagnetic tomographic methods is that the accurate reconstruction of electrical properties (e.g., conductivity) distribution around the interior of the target is challenging due to the lower sensitivity of measurements in its interior versus near the boundaries [25,26]. This limitation may cause inaccurate localization and quantification of defects and may be even more severe for open cell lattice structures. To solve this problem, Baltopoulos et al. [27] proposed reserving smaller singular value decomposition (SVD) components of the sensitivity map for efficient conductivity reconstruction of the center region by choosing a smaller hyperparameter, but this solution may introduce additional artifacts in the region of interest, hence deteriorating reconstruction quality. Li et al. [26] used a normalized sensitivity map to compensate for the low central sensitivity, but the proposed normalized methods are element-based and are computationally intensive. The element-based normalization is effective for detecting perturbations with large-area conductivity change. However, the method is not as effective for small-area defects, such as small defects in open cell lattice structures with small cross-section struts, where reconstructions would still suffer from image artifacts. These image artifacts may result in inaccurate defect detection or incorrect decisions. Thus, to improve reconstruction performance with respect to small perturbations, the normalized sensitivity map should be adjusted to be capable of compensating for low central sensitivity and restraining image artifacts.

In this work, we present a high-throughput, 3D, iterative, absolute conductivity distribution ERT system for identifying single and multiple damaged struts in conductive cellular lattice structures. The significance of this work is that the ERT algorithm employs a strut-based normalized map that preconditions the sensitivity map for enhancing conductivity reconstruction sensitivity while mitigating artifacts due to the ill-conditioning of the ERT inverse problem. The efficacy of this method was assessed by quantifying the relationship between damage severity and the corresponding reconstructed conductivity changes. Both numerical simulations and corresponding experiments of cellular lattice structures with different damage features were performed. To demonstrate that ERT could examine conductive cellular lattice structures, experiments were performed using 3D-printed polymer cellular lattice structures, which were then coated with a soluble, sacrificial, and electrically conductive nanocomposite thin film. Damage scenarios with single and multiple damaged struts were considered.

## 2. Theory and Methods

In this section, the theory of the absolute ERT method is introduced first. Then, the strut-based normalized sensitivity map and the quantitative defect detection method are discussed in detail.

### 2.1. ERT Theory

ERT is a nonintrusive soft-field imaging method that relies on boundary current injections and voltage measurements for reconstructing the conductivity distribution of a conductive body (or target) [28,29]. It is known that localized damage (e.g., voids, cracks, or broken parts) in the target can prevent or limit electric current propagation through that specific region. Therefore, identifying the magnitudes and locations of localized conductivity changes in reconstructions would enable direct visualization of damage severity and their respective locations. ERT consists of forward and inverse problems. The boundary voltages can be calculated by solving the forward problem based on the known conductivity distribution and the injected electrical current [22]. Practical implementations of ERT require solving the inverse problem, which reconstructs conductivity distribution from a set of experimental boundary potential measurements formed from unique current injection patterns [22]. 

In the forward problem, electric current propagation within a conductive target, Ω, with no interior current source, is governed by Laplace’s equation [22,28,30]:

(1) $$\nabla \cdot \left(\sigma \nabla \phi \right)=0\;\mathrm{in}\;\Omega$$

where *σ* is the conductivity distribution of Ω, and *ϕ* is the electric potential in the domain. The Dirichlet and Neumann boundary conditions and the complete electrode model are also defined in Equations (2)–(4), respectively [22,28]:

(2) $$\int_{e_{l}}^{}\sigma \frac{\partial \phi }{\partial n}dS=I_{l}\;\mathrm{on}\;\Gamma_{1}\;$$

(3) $$\sigma \frac{\partial \phi }{\partial n}=0\;\mathrm{on}\;\Gamma_{2}$$

(4) $$\phi +z_{l}\sigma \frac{\partial \phi }{\partial n}=V_{l}\;\mathrm{on}\;\Gamma_{1}$$

where *e_l_* is the location of the *l^th^* electrode, *n* is the normal direction from the boundary, *z_l_* is the contact impedance at the *l^th^* electrode, and *I_l_* and *V_l_* are the injected current and electric potential on the *l^th^* electrode, respectively. Here, Γ_1_ is the boundary with electrodes, while Γ_2_ refers to the remainder of the boundary. Equations (1)–(4) are numerically solved by the finite element (FE) method with a known *σ* distribution for simulated voltage responses *V*(*σ*) [22,28].

The inverse problem aims to reconstruct the interior conductivity distribution of Ω by minimizing the difference between experimentally measured boundary voltages (*V_m_*) and simulated voltages *V*(*σ*), starting with an assumed conductivity distribution *σ*. The objective function (*g*) is added with a Tikhonov regularization term as Equation (5) and solved with the Gauss-Newton iterative algorithm due to the ill-posed nature of the ERT inverse problem [28,31]:

(5) $$g=\underset{\sigma }{\min}\{||V_{m}-V\left(\sigma \right){||}^{2}+\lambda ||R\sigma {||}^{2}\}$$

where *λ* is the hyperparameter, and *R* is a regularization matrix. The algorithm iteratively updates the reconstructed conductivity distribution with Δ*σ^i^*^+1^ in each (*i*+1)*^th^* iteration as:

(6) $$\Delta \sigma^{i+1}={\left(J^{i}{}^{T}J^{i}+\lambda R^{T}R\right)}^{-1}\cdot J^{i}{}^{T}\cdot \left[V_{m}-V\left(\sigma^{i}\right)\right]$$

(7) $$J^{i}=-\int_{\Omega }^{}{\left(\nabla \phi_{p}^{i}\right)}^{T}\nabla \phi_{q}^{i}dx^{3}$$

where the sensitivity map, *J^i^*, is the derivative of *V*(*σ^i^*), and $\phi_{p}^{i}$ and $\phi_{q}^{i}$ are the nodal electric potentials considering a current passing through the pair of current injection electrodes and voltage measurement electrodes, respectively [31,32,33]. The reconstruction process continues until the error ratio, which is defined as the norm of the difference between *V*(*σ^i^*) and *V_m_* normalized by the norm of *V_m_*, is not improving by 0.1% for the following iterations, then returns the final reconstructed conductivity distribution, *σ_r_* [22].

### 2.2. Adjusted Absolute Imaging

**Figure 1:**
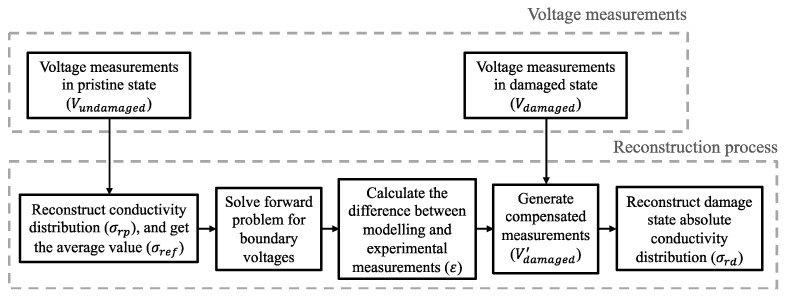
The flow chart illustrates the adjusted absolute imaging process.

Solving the ERT inverse problem with Equation (6) is referred to as absolute imaging, which reconstructs absolute conductivity distribution. However, in practical ERT implementations, errors from measurements, inaccuracies from spatial inhomogeneity, and the modeling of electrode positions could affect the accuracy of the reconstruction result of the target in the damaged state when using absolute imaging directly [34]. In this study, we employ an adjusted absolute imaging method that efficiently compensates for those errors, and the workflow is illustrated in Figure 1. This method calculates the modeling error (*ε*) between experimental undamaged state measurements (*V_undamaged_*) and the voltages *V*(*σ_ref_*) calculated by the assumed homogenous model, before locally subtracting them from damaged state measurements (*V_damaged_*), as is shown in Equations (8) and (9). The undamaged state measurements usually could be easily obtained from itself or other qualified structures in a mass production [29,34]. The updated measurements (*V*′*_damaged_*) compensate for modeling inaccuracy and are directly used to reconstruct the absolute conductivity distribution (*σ_rd_*) of the target. This mechanism enhances reconstruction quality by transforming the inverse problem from a global to a local minimization process [34].

(8) $$\varepsilon =F\left(\sigma_{ref}\right)-V_{undamaged}$$

(9) $$V\prime_{damaged}=V_{damaged}-\varepsilon$$

### 2.3. Modification of the Sensitivity Map

In ERT, reduction of sensitivity in the target’s central causes relatively low reconstructed conductivity changes and more image artifacts, especially considering measurement noises [26,35]. To address this limitation, a strut-based normalization procedure is proposed and can be imposed on the sensitivity map to improve *σ* reconstruction.

**Figure 2:**
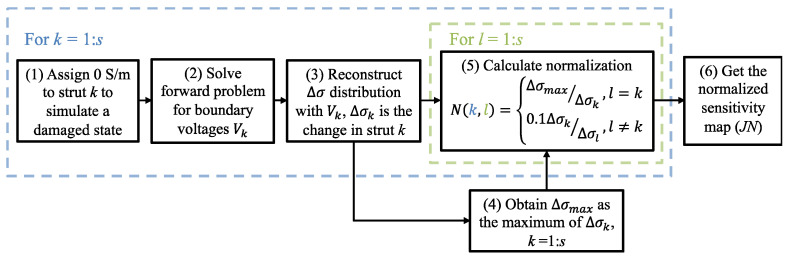
The flow chart illustrates the calculation of the normalized sensitivity map.

Figure 2 outlines the procedure for the strut-based normalization process, where the objective is to obtain uniform boundary-voltage-to-conductivity-perturbation sensitivity in each strut (i.e., regardless of their location in a cellular lattice structure). To calculate the normalized sensitivity, the reconstruction results from classical ERT for each damaged state (where a damaged strut *k* is assigned with 0 S/m) are obtained first in steps (1) to (3). A total of *s* damaged states are solved considering the total of *s* number of struts in the structure. Among all states, the largest reconstructed change within the damaged strut (Δ*σ_max_*) could be acquired in step (4) when damage is assigned on the boundary strut. During the calculation of the strut-wise normalization matrix (*N*) in step (5), diagonal components of the matrix *N* are normalized to compensate for relatively low responses in the central struts, and the non-diagonal components are used for suppressing artifacts to 0.1*σ_k_*, which would not affect the defect evaluation. With this normalization, the adjusted sensitivity map could be obtained as *JN* in step (6), and the change in voltage measurements corresponding to a single perturbation would be:

(10) $$\delta V=\left(JN\right)\left(N^{-1}\right)\delta \sigma$$

The results, which show the benefits of using the normalized sensitivity map to improve interior sensitivity and mitigate image artifacts, will be discussed in Section 3 and Section 4.

### 2.4. Representative Strut Conductivity and Defect Quantification

**Figure 3:**
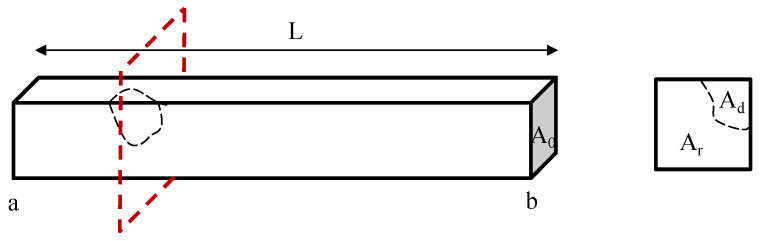
Damage that was introduced in a strut is illustrated, and the strut representative (*σ_s_*) conductivity could be further calculated.

Damage severity within each strut can be reflected by a single index, which is referred to in this study as strut representative conductivity, *σ_s_*. Here, *σ_s_* is the equivalent conductivity of a damaged strut, which is calculated using the electric potential drop between the two ends of the strut, *V_ab_*, while assuming its dimensions remain the same. *V_ab_* is affected by the size, shape, and amount of damage developed in the strut. With known damage features shown in Figure 3, *σ_s_* could be calculated as:

(11) $$V_{ab}=\int_{0}^{L}\frac{I}{A_{r}\sigma_{0}}dl$$

(12) $$\sigma_{s}=\frac{IL}{V_{ab}A_{0}}=\frac{L}{A_{0}\int_{0}^{L}\frac{1}{A_{r}\sigma_{0}}dl}$$

where *σ*_0_ is the material conductivity in its undamaged state, *L* is the length of the strut, and *A_r_* is the residual area (i.e., the cross-sectional area where the defected region *A_d_* is subtracted from the undamaged cross-section *A*_0_) for a differential length, *dl*. 

During ERT inspection, defects in struts with small cross-sectional areas could not be effectively localized given the limited resolution and electric field propagation pathways in topologically ordered open cell structures. However, the reconstructed conductivity (*σ_r_*) in each strut could be used to examine damage severity. Here, *σ_s_* which corresponds to the damage could serve as a comparison parameter with respect to *σ_r_* solved by ERT with an invariant struts model. In this study, *σ_r_* was compared with *σ_s_* in both simulations and experiments to validate the quantitative defect detection capabilities of the proposed ERT method.

## 3. Simulation Details and Results

### 3.1. 3D ERT Numerical Simulations

**Figure 4:**
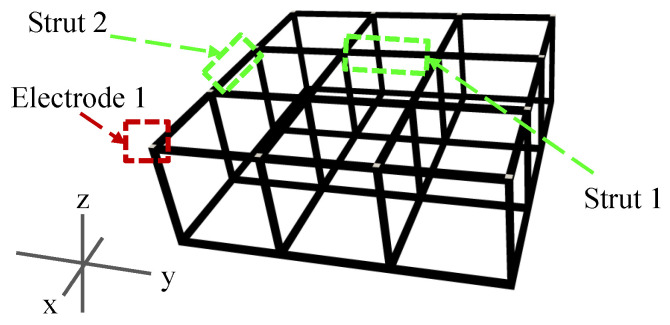
A 3 × 3 × 1 lattice structure model was created in Abaqus. Electrodes in the upper z-plane are marked in white.

The feasibility of 3D ERT for detecting and localizing damaged struts in cellular lattice structures was first assessed with numerical simulations. A 3 × 3 × 1 lattice structure with cubic unit cells consisting of 40 mm long and 2 × 2 mm^2^ cross-section struts was constructed in Abaqus, as is shown in Figure 4; the cellular lattice structure was meshed using 9229 tetrahedral elements. Electrodes were defined at the 24 intersecting nodes along the boundaries. The conductivity of all the elements was assumed to be 1000 S/m, based on the resistance measurements of the CNT thin film coat used in the following experiments.

A quantitative damage assessment study was performed by executing the 3D ERT forward and inverse algorithms on the undamaged lattice structure, as well as on assumed single-defect cases. Defect severity was simulated by considering two defect propagation situations in an interior strut (strut 1), where the size of the damage feature could grow along the length or depth of the strut. Damage propagating along the strut length was modeled by assigning 0 S/m to adjacent finite elements in the longitudinal direction of the initial damage site, while damage propagating along depth considered 0 S/m elements in the transverse direction and along the strut cross-section. A fully damaged strut was simulated by assigning 0 S/m to all elements along the strut cross-section (i.e., the strut is completely broken). 

To simulate a multi-defect damage case, an additional full strut length breakage was then introduced in strut 2 by assigning all finite elements within the strut to be 0 S/m. For each undamaged and damaged scenario, the 3D ERT forward problem was executed by applying direct current (DC) between all adjacent electrode pairs (i.e., adjacent current injection pattern) on each z- or 3 × 3 plane (Figure 4). The complete set of 504 boundary voltages calculated from the forward problem were corrupted with Gaussian white noise signal with a signal-to-noise ratio (SNR) of 66.2 dB, considering the measured SNR is between 65 dB and 68 dB and simulations conducted by Polydorides et al. [36]. The voltage dataset was then used as the input for the ERT inverse solver to reconstruct the 3D conductivity distribution of the lattice structure model.

### 3.2. Sensitivity Discussion

**Figure 5:**
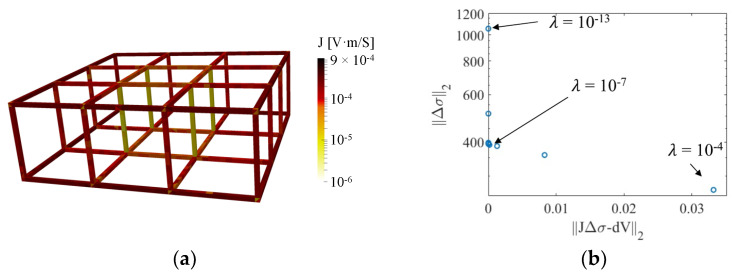
(**a**) The summed sensitivity map of the lattice structure was calculated. (**b**) The *L*-curve was plotted, with *λ* ranging from 10^−13^ to 10^−4^.

The sensitivity map relates the conductivity perturbation of each finite element to the corresponding variations in boundary electrode voltages. The magnitude of sensitivity is correlated to electric field propagation induced by current injected in a pair of boundary electrodes [33]. In general, the electric field in the center of the target would be much lower than near the boundary, resulting in lower sensitivity in the center. Figure 5a plots the summation of the absolute values of sensitivity for each finite element when the lattice structure was interrogated using the adjacent injection pattern (in logarithmic scale). The bright color in the central struts illustrates the decreased sensitivity at the center. Because of the in-plane current injection scheme, the sensitivity of vertical struts along the z-axis is lower than the in-plane struts. The hyperparameter in the inverse problem controls the number of valid SVD components of the sensitivity map. Usually, choosing a smaller hyperparameter will reserve more small SVD components and improve reconstructions of central conductivity changes [27]. However, it is difficult and inefficient to select the appropriate hyperparameter regarding conductivity perturbation happening in different regions of an open cell lattice structure. 

Therefore, instead of adjusting the hyperparameter for different conductivity perturbation situations, a normalized sensitivity map was implemented. In accordance with the uniform normalized sensitivity map, the hyperparameter for a uniform conductivity perturbation in the entire region was chosen with the *L*-curve method for the reconstruction process [27]. In this case, the *L*-curve with the hyperparameter ranging from 10^−13^ to 10^−4^ is plotted in Figure 5b, and 10^−7^ (near the inflection point) was chosen.

### 3.3. Assessment of Conductivity Reconstruction

Classical image evaluation criteria and an additional quantitative criterion were employed to evaluate the conductivity imaging performance of ERT with and without the normalized sensitivity map. It should be clarified that the scope of this study only considered damaged struts, so only strut-based errors were evaluated. The image evaluation criteria of position error *e_C_* and area error *e_A_* are calculated as:

(13) $$e_{C}=\frac{\left|C_{r}-C_{s}\right|\;}{L_{p}}$$

(14) $$e_{A}=\frac{\left|A_{r}-A_{s}\right|}{A_{p}}$$

where *C_s_* and *A_s_* are the centroid and damage area of the real damaged strut, respectively, while *C_r_* and *A_r_* are the reconstructed damage centroid and damage area, respectively, which are defined by conductivity changes larger than one-fourth of the maximum conductivity change [37,38]. The undamaged strut’s length *L_p_* and area *A_p_* are included for normalization. In addition, the reconstructed error value, *e_σ_*, is defined to assess the difference between reconstructed conductivity (*σ_r_*) and the calculated strut representative conductivity (*σ_s_*) normalized by undamaged state conductivity *σ_p_*.

(15) $$e_{\sigma }=\frac{\left|\sigma_{r}-\sigma_{s}\right|}{\sigma_{p}}$$

### 3.4. Single-Defect Detection

**Figure 6:**
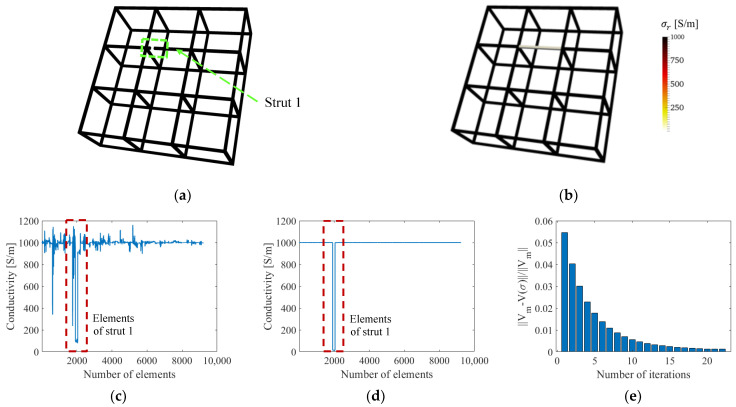
(**a**) A lattice structure was imposed with damage. (**b**) An ERT reconstruction was solved with the normalized sensitivity map. (**c**) The reconstructed conductivity values of each element when solved without and (**d**) with the normalized sensitivity map are plotted. (**e**) The normalized errors are plotted with iterations.

Different damage severities were imposed in the single-defect case, and only the reconstructed conductivity values were affected but not the localization of the defect. Thus, the single-defect case with a fully damaged strut (Figure 6a) was investigated and reconstructed by ERT (Figure 6b) first. The ERT conductivity distribution of the single-defect lattice structure was reconstructed without and with the normalized sensitivity map; plots of reconstructed conductivity values with respect to the finite elements are shown in Figure 6c,d, respectively. A total of 22 iterations were conducted in the inverse process with the normalized sensitivity map to reach the error ratio tolerance. The decrease of the error ratio is shown in Figure 6e. Classical ERT (i.e., without the normalized sensitivity map) could not accurately reconstruct the conductivity value (i.e., 0 S/m) of the central damaged strut but instead could only approach it (i.e., 96 S/m), as is shown in Figure 6c. The reconstructed conductivities in the undamaged struts also show significant variations and deviate from the true value of 1000 S/m. In contrast, Figure 6d shows that the reconstructed conductivity values when using ERT with the normalized sensitivity map were similar to the actual case (i.e., either 1000 or 0 S/m). In fact, the corresponding strut-based image errors *e_C_* and *e_A_* are all zeros as shown in Table 1. The improved reconstruction performance occurred because normalization compensates for the low central region sensitivity by imposing corresponding weighting factors that facilitated accurate conductivity reconstruction. 

**Figure 7:**
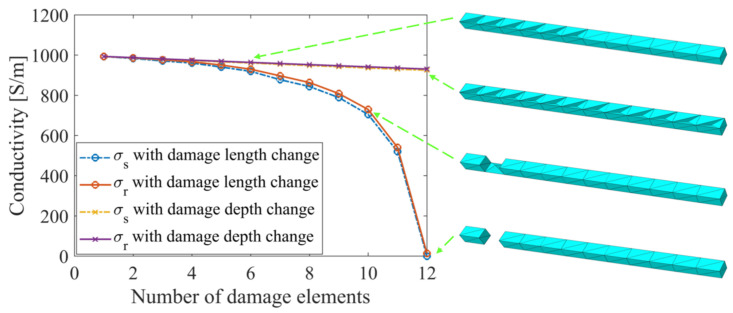
Reconstructed conductivity in the strut *σ_r_* is consistent with representative strut conductivity *σ_s_*. Depth and length of the damage feature are varied by assigning 0 S/m to *n* finite elements.

As more accurate reconstructed conductivity values were achieved by using ERT with the normalized sensitivity map, the quantitative defect detection ability was further examined with results solved with the normalized sensitivity map. A total of 24 different assumed single-defect cases considered two defect propagation situations were discussed, either along the length or depth of the strut. The first set of 12 damage cases considered a single crack propagating longitudinally in strut 1, where different damage scales (which were defined by damage width and length) were simulated by assigning a conductivity of 0 S/m to n longitudinally adjacent finite elements. The second set of 12 damage cases were introduced on another undamaged structure, with a defect propagated transversely in strut 1 by imposing 0 S/m on n elements along the strut cross-section. Up to 12 elements were assigned with 0 S/m to simulate a fully damaged strut. In Figure 7, the change of the reconstructed conductivity (*σ_r_*) within the damaged strut is consistent with the strut representative conductivity (*σ_s_*) for both imposed damage propagation scenarios. Their consistency expresses the significance of calculating strut representative conductivity and the capability of the ERT method with the normalized sensitivity map to return conductivity values corresponding to the damaged states. The reconstructed errors (*e_σ_*) of the damage cases with defect propagation along depth are shown in Table 2. The trends shown in Figure 7 demonstrate that the reconstructed values are more sensitive to damage propagated in the transverse direction (as opposed to the longitudinal direction), as suggested by Equation (10).

### 3.5. Multi-Defect Detection

**Figure 8:**
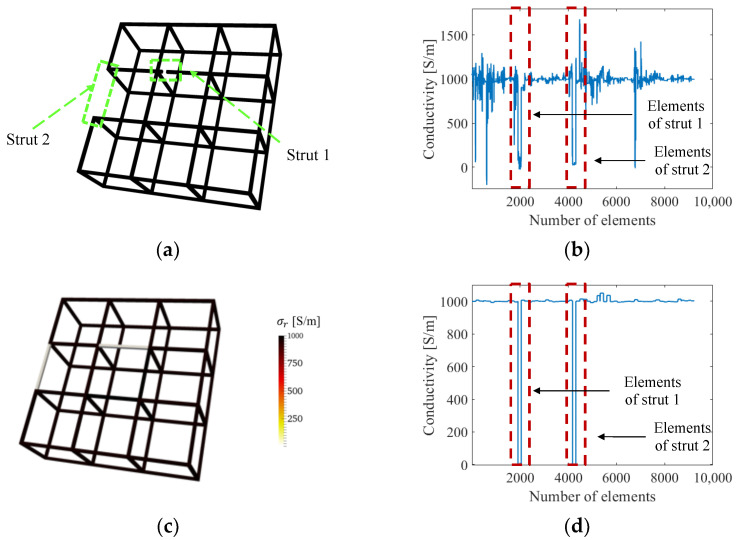
(**a**) A lattice structure with two damaged struts. (**b**) The reconstructed conductivity values of each element when solved without the normalized sensitivity map. (**c**) The reconstructed 3D conductivity distribution and (**d**) the conductivity values for each element, when solved using the normalized sensitivity map.

We also considered multiple defect sites by introducing an additional full strut breakage to a boundary strut (strut 2), as shown in Figure 8a. The ERT result solved without the normalized sensitivity map in Figure 8b was littered with artifacts, and *e_σ_* of strut 1 (see Table 3) is approximately twice that of strut 2, because strut 2 is closer to the boundary electrodes. In contrast, Figure 8c,d show the reconstructed conductivity distribution in 3D visualization and with respect to finite elements, respectively, when using the normalized sensitivity map. In addition to significantly reducing conductivity reconstruction artifacts, normalization yielded uniform sensitivity throughout the cellular lattice structure. The reconstructed conductivity for both damaged struts approaches 0 S/m and can be clearly interpreted as breakages, as can be seen in Figure 8d, and both error values are 20 times lower than the case without normalization. Overall, these simulation results demonstrated improved spatial and quantitative accuracy when the normalized sensitivity map is incorporated with ERT. 

### 3.6. Defect Detection in Complex Lattice Structures

**Figure 9:**
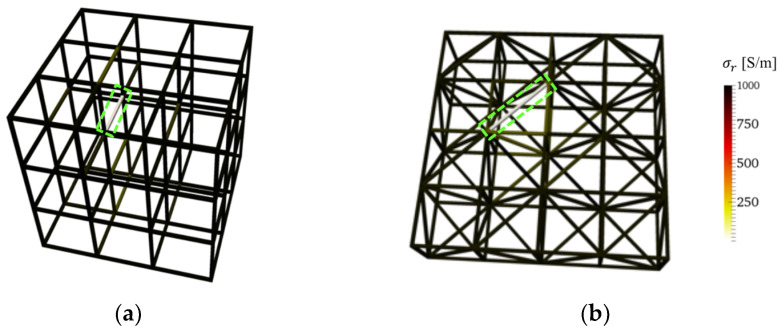
3D conductivity distribution reconstructions of (**a**) 3 × 3 × 3 and (**b**) 4 × 4 × 1 (with diagonal struts) lattice structures successfully identified the broken strut in each structure.

The effectiveness of this method was validated by considering full strut breakages in other complex lattice structures. Simulations were conducted using a 3 × 3 × 3 lattice with cubic unit cells, as well as a 4 × 4 × 1 lattice structure with diagonal struts. Similar to previous cases, boundary electrodes were defined as the intersecting nodes along the boundaries. For the 3 × 3 × 3 lattice, only the top and bottom faces (i.e., top and bottom z-planes) had electrodes for a total of 24 boundary electrodes. On the other hand, the 4 × 4 × 1 lattice had 32 boundary electrodes. Broken struts (i.e., where conductivity is 0 S/m) were defined in various locations in the lattice structures. Similar to Section 3.4, the ERT method with the normalized sensitivity map was employed to reconstruct the conductivity distribution of the two structures, which are shown in Figure 9a,b. The results confirmed that the ERT solver was able to correctly identify the simulated broken struts.

## 4. Experimental Details and Results

### 4.1. 3D-Printed Lattice Structures

**Figure 10:**
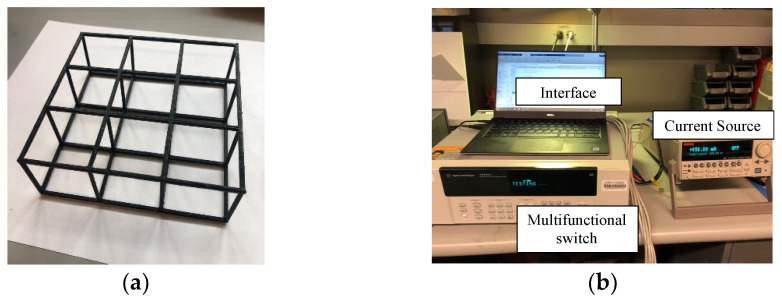
(**a**) A 3 × 3 × 1 lattice structure was spray-coated with a conductive, nanocomposite thin film. (**b**) ERT measurements were obtained using a customized data acquisition system.

Experiments were performed on 3D-printed cellular lattice structures to validate damage detection and localization (Figure 10a). A commercial fused deposition modeling (FDM) Ultimaker 3+ 3D-printer (Ultimaker, Utrecht, Netherlands) fabricated 3 × 3 × 1 polylactide acid (PLA) lattice structures with cubic unit cells identical to the structure described in Section 3.1. The PLA lattice structure was coated with a multi-walled carbon nanotube (MWCNT) thin film. First, a paint primer layer was spray-coated onto the lattice structure. Second, an MWCNT-latex ink was prepared following the procedure described by Mortensen et al. [39] and Wang et al. [40]; MWCNTs were purchased from SouthWest NanoTechnologies (Norman, OK, USA). Lastly, upon complete air-drying of the nanocomposite in ambient conditions, 24 boundary electrodes were formed by drying colloidal silver paste (Ted Pella, Redding, CA, USA) over conductive threads (Adafruit, New York, NY, USA) at the intersecting boundary nodes, without damaging the structure.

### 4.2. 3D ERT Data Acquisition and Testing

The customized 3D ERT data acquisition (DAQ) system employed in this study is shown in Figure 10b. It consists of a Keysight 34980A multifunctional switch (with an internal digital multimeter, Keysight, Santa Rosa, CA, USA) and a Keithley 6221 current source (Cleveland, OH, USA), which were connected and controlled by MATLAB. The current source was commanded to inject 10 mA of DC to an adjacent pair of boundary electrodes, while the switch sequentially measured and recorded 504 boundary voltage measurements. The same adjacent electrode method reported in Section 3.1 was utilized to inspect the nanocomposite-coated lattice structure. 

Three sets of cases were considered: (1) undamaged state, (2) single-defect damaged state, and (3) multi-defect damaged state. First, the undamaged 3D lattice structure was interrogated to reconstruct the undamaged state conductivity distribution of the test specimens. Second, seven different single-defect damage cases (Cases #1 to #7 in Table 4) were prepared and tested. Each of these cases featured one damaged internal strut, where damage was introduced by mechanically etching off a portion of the film on the damaged strut. Table 4 shows how the single-defect damage cases were unique. The film was removed from one to four of the faces of the square-cross-section strut, while the length of the damage varied between *L*/4 to *L*, where *L* is the total length of the strut. In particular, Case #7 corresponded to the case when the film was removed from the entire strut, so the electric current could not flow through the strut (i.e., to emulate complete strut breakage). Lastly, the multi-defect damaged state, Case #8, considered two damaged struts with the nanocomposite completely removed. 

### 4.3. Single-Defect Detection

**Figure 11:**
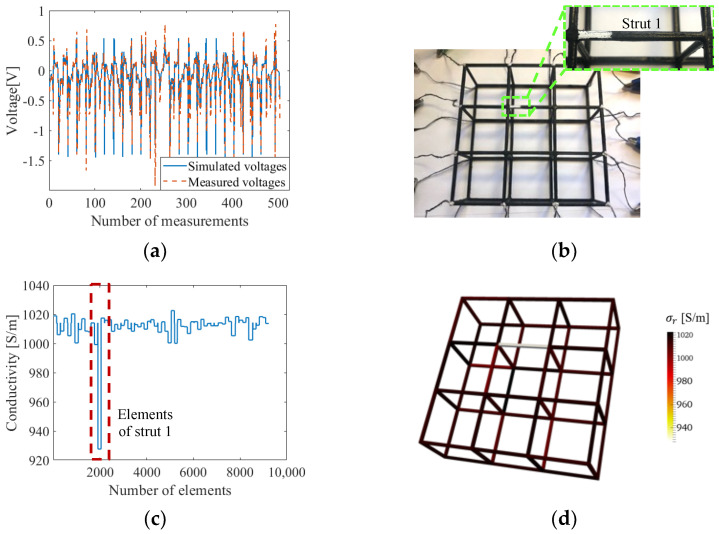
(**a**) Simulated voltages are compared with experimentally measured voltages. (**b**) The first etch (damage) was introduced in the lattice. (**c**) The reconstructed conductivity values of each element when solved with the normalized sensitivity map are plotted. (**d**) The corresponding 3D conductivity distribution successfully confirmed damage detection in strut 1.

Experimental realization of the ERT method depends on the robustness of the experimental data as well as modeling accuracy, where accuracy can be impaired by data with environmental noise and inaccurate modeling. We hypothesized that the adjusted absolute imaging used in this study could diminish errors by compensating modeling errors with local minimization. An example of a direct comparison between simulated and experimentally measured boundary voltages is shown in Figure 11a and confirms the degree of mismatch was minor.

The defect detection performance of the ERT system with the normalized sensitivity map was examined with experimental measurements in Case #1. The picture of Figure 11b shows that the film was etched off on the upper side of strut 1 with a total etched length of *L*/4. From the results obtained from 36 iterations in the inverse process and evaluations shown in Figure 11c and Table 5, artifacts were restrained to some extent with the application of the normalized sensitivity map. These minor conductivity artifacts were the result of experimental measurement noise and modeling inaccuracies of modeling. The reconstructed value of 928 S/m in strut 1 is related to the size of the etch and will be discussed more in the quantitative study. The reconstructed conductivity distribution of the lattice structure with a single damaged strut is visualized in Figure 11d.

**Figure 12:**
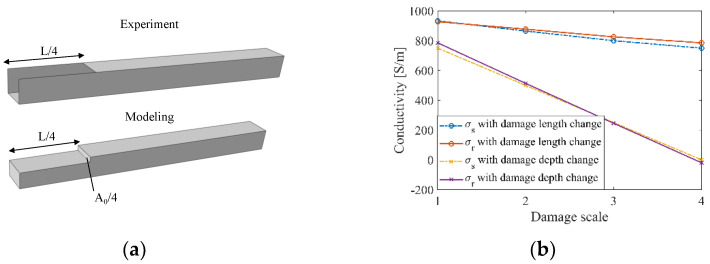
(**a**) The actual experimental damage (Case #1) was compared to what was modeled. (**b**) Representative strut conductivity *σ_s_* and reconstructed conductivity in the strut *σ_r_* change in tandem as damage increased in severity, both along its length and depth (cross-section).

The quantitative damage detection performance of ERT was evaluated with experimental measurements in the single-defect damaged state, from Cases #1 to #7. Although only conductive nanocomposites were coated onto PLA lattice structures, the ERT FE model still considered solid struts, because the modeling inaccuracy of strut cross-sections is admissible due to the strut-wise defect detection capability of ERT on lattice structures. This meant that actual damage, such as film etched off a single face, was modeled as a one-fourth cross-section reduction. Thus, Case #1 was assumed to have experienced a volume reduction of one-fourth *A*_0_ and one-fourth *L*, as illustrated in Figure 12a. The reconstructed image and value were then evaluated with the representative model and strut representative conductivity (*σ_s_*), which was calculated using Equations (9) and (10).

The conductivity results of Cases #1 to #7 (with etches of different sizes along the length and different faces) were reconstructed using ERT with the normalized sensitivity map. Among these, Cases #1 to #4 considered damage occurring on a single face but increased in length from *L*/4 to *L* (i.e., similar to damage propagating along the length of the strut). Cases #5 to #7 corresponded to damage growing in depth. The comparison between reconstructed conductivities solved with the normalized sensitivity map and the strut representative conductivities is presented in Figure 12b, while the calculated errors listed in Table 6 show their consistency. From Figure 12b, it can be seen that damage along the cross-section of the strut can be detected at a higher sensitivity than those along the length. Overall, the similarity between the reconstructed and strut representative conductivity results experimentally validated the proposed ERT method. 

### 4.4. Multi-Defect Detection

**Figure 13:**
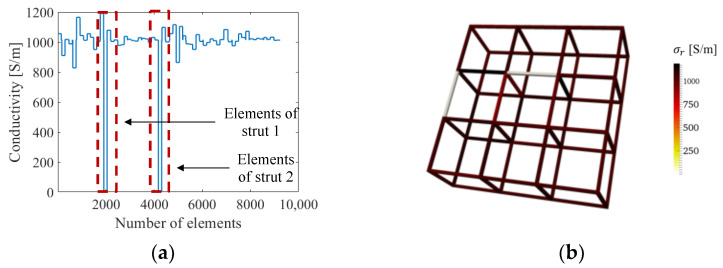
(**a**) The reconstructed conductivity values of each element when solved with the normalized sensitivity map are plotted. (**b**) The corresponding 3D conductivity distribution of the lattice structure successfully identified breaks in strut 1 and strut 2.

In addition to the single-defect scenarios (i.e., Cases #1 to #7), Case #8 with an additional broken strut (i.e., strut 2) was considered. The conductivities of each element reconstructed using ERT with the normalized sensitivity map are plotted in Figure 13a. Similar to the previous results, damage in the broken struts could be identified, and the conductivity values approached 0 S/m. Artifacts were present in other elements, but their magnitudes are at least 83% lower than those corresponding to the two broken struts, which are also evident based on the calculated error values listed in Table 7. Because these correspond to experimental results, artifacts due to measurement noise and mismatch between simulation modeling and experiment are inevitable. Nevertheless, the values of *e_σ_* in Table 7 clearly show that ERT with the normalized sensitivity map outperforms classical ERT. 

## 5. Discussion

The simulation and experimental results showed that the 3D ERT method with the strut-based normalized sensitivity map was able to characterize damage accurately and quantitatively in cellular lattice structures. The strut-based normalized sensitivity map compensated for the low central sensitivity and drastically reduced image artifacts, so the reconstructions had much smaller reconstruction errors as defined by *e_C_*, *e_A_*, and *e_σ_*.

However, it is worth mentioning that the implementation of ERT in practice may face certain challenges, since electrodes need to be physically attached to the structure for propagating electrical current. Improperly attached electrodes can introduce unwanted contact impedance (especially if electrodes are not permanently mounted) and subsequently affect the reconstructed conductivity distributions. A potential solution is to use spring-loaded press-contact electrodes that can apply a consistent force at each electrode during ERT interrogation and measurements. On the other hand, extreme or varying ambient temperatures and environmental conditions can also potentially affect the conductivity of the structure and thus the recorded boundary voltages. Besides leveraging reference sensors that quantify these ambient effects, another approach can be optimizing the electrode configuration so that the minimum number of electrodes and measurements are needed to achieve the desired damage quantification resolution. Fewer electrodes and measurements mean that ERT interrogation can be performed faster, and varying ambient effects become less significant. 

Overall, the 3D ERT method is an efficient method for detecting damage in lattice structures. With only a few electrodes attached to the boundary and their corresponding voltage measurements, the resistivity distribution that correlated to the damaged state could be captured. Currently, vibrational-based methods could only offer classification of different damage scenarios but could not effectively pinpoint specific damaged struts unlike the 3D ERT method [41]. Moreover, ERT utilizes the intrinsic electromechanical properties of lattice structures and renders effective inspection by propagating current throughout the entire structure. Furthermore, X-ray CT-based measurements or other image processing methods require the structure to be placed between a source and detector while being rotated to obtain multiple projection slices, which requires extensive operational times and computational resources [42].

## 6. Conclusions

Here we demonstrate an absolute, high-performance, 3D ERT method which incorporates a strut-based normalized sensitivity map for quantitative defect detection in lattice structures with high image accuracy. The approach was applied for detecting multiple defects in open-cell lattice structures. The strut-based normalized sensitivity map addressed the issue of heterogeneous damage sensitivity, particularly lower sensitivity away from the boundaries where measurements are obtained. Simulations and experiments validated the improved defect detection capability of this method compared to classic ERT. In simulations, single- and multi-defect cases were realized by assigning 0 S/m to finite elements while the damage in experiments was realized by etching coated conductive nanocomposite thin films. Our results show that the ERT method with the normalized sensitivity map could localize defects more accurately and with smaller image errors compared to classical ERT. Quantitative damage detection performance was demonstrated by the strong consistency between reconstructed conductivity within a strut and the actual damage severity. Future work will examine the development of an electrical impedance tomography system to leverage alternating current input excitations for higher resolution defect imaging, and non-iterative reconstruction algorithms that not requiring a baseline measurement for high-speed anomalies detection instead of high-accurate conductivity reconstructions.

## Figures and Tables

**Table 1:** Image errors quantification and comparison of single-defect reconstructions in simulation.

Strut-Based Evaluation	*e_C_*	*e_A_*
Without normalized sensitivity map	0.0030	0.1314
With normalized sensitivity map	0	0

**Table 2:** Reconstructed value errors of single-defect reconstructions in simulation.

Damage Scale	*e_σ_*	Damage Scale	*e_σ_*
**1**	0.0008	**7**	0.0192
**2**	0.0026	**8**	0.0198
**3**	0.0052	**9**	0.0198
**4**	0.0060	**10**	0.0251
**5**	0.0100	**11**	0.0209
**6**	0.0105	**12**	0.0137

**Table 3:** Errors quantification and comparison of multi-defect reconstructions in simulation.

Strut-Based Evaluation	*e_C_*	*e_A_*	*e_σ_* of Strut 1	*e_σ_* of Strut 2
Without normalized sensitivity map	0.0146	0.1528	0.0752	0.0329
With normalized sensitivity map	0	0	0.0032	0.0017

**Table 4:** Summary of experimental test cases.

	Single-Defect							Multi-Defect
Case	#1	#2	#3	#4	#5	#6	#7	#8
Number of damaged struts	1	1	1	1	1	1	1	3
Number of damaged faces	1	1	1	1	2	3	4	8
Total damaged length	*L*/4	*L*/2	*3L*/4	*L*	*L*	*L*	*L*	2*L*

**Table 5:** Image errors comparison of single-defect reconstructions in experiment.

Strut-Based Evaluation	*e_C_*	*e_A_*
Without normalized sensitivity map	0.0696	0.1551
With normalized sensitivity map	0	0

**Table 6:** Reconstructed value errors of single-defect reconstructions in experiment.

Damage Case	#1	#2	#3	#4	#5	#6	#7
*e_σ_*	0.0053	0.0199	0.0257	0.0353	0.0134	0.0042	0.0244

**Table 7:** Errors quantification and comparison of multi-defect reconstructions in experiment.

Strut-Based Evaluation	*e_C_*	*e_A_*	*e_σ_* of Strut 1	*e_σ_* of Strut 1
Without normalized sensitivity map	0.0287	0.2233	0.0653	0.0804
With normalized sensitivity map	0	0	0.0056	0.0030

## Data Availability

Not applicable.
